# The Elemental Composition of Land Plants: A Global Database and Meta-analysis

**DOI:** 10.1021/acsenvironau.6c00028

**Published:** 2026-07-23

**Authors:** Harrison R. Coker, Aenghus C. Denvir, Amir M. Mokhtari, Anna Lewkowicz, Patrick Grove, Kristina Mikhailova, Colin Lennox, Marina López-Pozo, Artemi Jaumà-Palomeras, Luke G. Whiteley, Rachel C. Rivero, Borja Barbero Barcenilla, Julie A. Howe

**Affiliations:** † Department of Soil and Crop Sciences, Texas A&M University, Texas A&M AgriLife Research, College Station, Texas 77843, United States; ‡ Department of Developmental Biology, Institute of Dendrology, Polish Academy of Sciences, Kórnik 62-036, Poland; § Department of Environmental Systems Science, ETH Zürich, Zurich 8092, Switzerland; ∥ The Spring Institute for Forests on the Moon, Pleaux 15700, France; ⊥ Department of Chemistry and Chemical Biology, 5922 Cornell University, Ithaca, New York 14853, United States; # EcoIslands LLC, Altoona, Pennsylvania 16601, United States; ¶ Department of Ecology and Evolutionary Biology, 1877 University of Colorado, Boulder, Colorado 80309, United States; ∇ 16767 Universitat Politècnica de Catalunya, Barcelona 08034, Spain; ○ Department of Molecular, Cellular, and Developmental Biology, 1259 University of Michigan, Ann Arbor, Michigan 48109, United States; ⧫ Department of Biochemistry and Biophysics, 14736 Texas A&M University, College Station, Texas 77843, United States

**Keywords:** plant, nutrition, mineral, database, global, fertility

## Abstract

Although plants likely take up all elements present in the soil environment, the elemental composition of land plants remains poorly characterized beyond essential nutrients. A comprehensive global database of plant tissue concentrations spanning 52 elements was compiled from 5,474 samples across 73 countries, 21 climate classes, and 26 soil groups. Robust global means for macronutrients, micronutrients, transition and heavy metals, metalloids, and rare earth elements are reported using mixed-effects models that account for study level variation. Global concentrations are also reported for roots and shoots, domesticated and wild plants, edible and nonedible plants, N-fixation symbiosis, and for various plant types including trees, shrubs, vines, forbs, sedges, grasses, ferns, and mosses. Many nonessential elements (i.e. transition metals, rare earth elements) exist in plant tissues in higher concentrations than micronutrients, with 20 elements higher than Mo. Phylogenetic analyses across major plant lineages revealed strong evolutionary constraints on elemental composition, with Ornstein–Uhlenbeck models indicating stabilizing selection toward lineage-specific optima for most elements. The plant elements most likely to predict taxonomic grouping through unsupervised machine learning (XGBoost) followed S > Ca > N > Zn > Ba > Cu > Pb > Sb > Cd > P. Multivariate ordinations separated domesticated crops from wild species, driven primarily by macronutrient enrichment in crops. Further, key atomic ratios differed between domesticated and wild plants, driven by differences in Zn:Cd, K:Na, N:S, and N:P. Systematic hyperaccumulator identification revealed 53 species across diverse taxa, with high Ni, Cu, and Pb uptake as the most prevalent indicator of hyperaccumulation. The study provides reliable and comprehensive estimates of the elemental composition of land plants and highlights that lesser studied elements constitute a considerable fraction of plant tissues.

## Graphical abstract

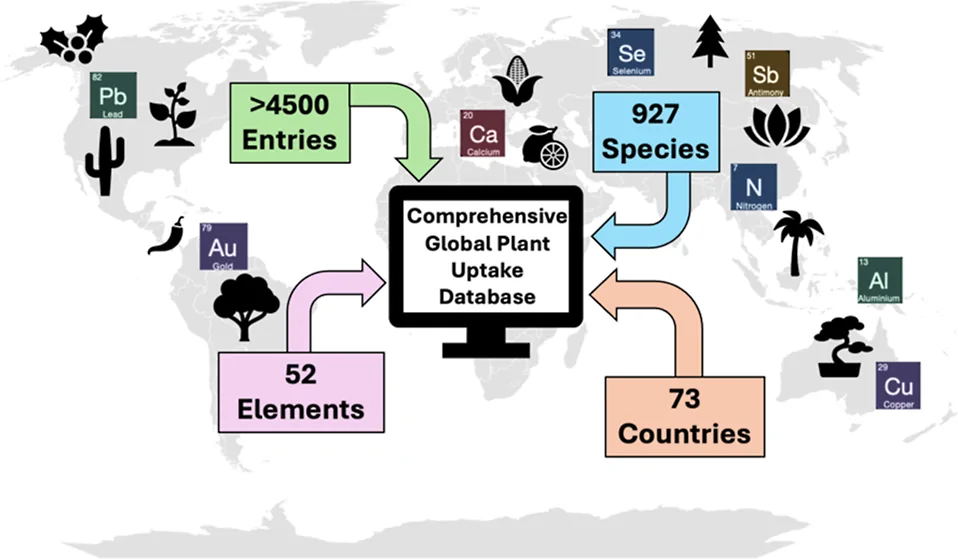

## Introduction

Plant mineral nutrition encompasses the uptake, transport, and utilization of elements essential for plant growth, metabolism, and reproduction.^1^ Traditional approaches to plant nutrition have focused primarily on macronutrients (N, P, K, Ca, Mg, and S) and micronutrients (Fe, Zn, Mn, Cu, Mo, and B), reflecting their economic importance and established roles in plant physiology. However, while there are 17 elements considered essential for plant productivity (C, H, O, N, P, K, Ca, Mg, S, B, Fe, Zn, Mn, Ni, Cu, Cl, and Mo), plants will likely take up all elements present in a mineral soil. A consideration of the comprehensive set of all elements^2^ that plants take up is fundamental to biogeochemical modeling, ecosystem management, and agricultural sustainability. Indeed, the discipline of biogeochemistry examines how elements cycle through the atmosphere, hydrosphere, biosphere, and lithosphere, with plants serving as critical intermediaries in these processes.^3^ Without comprehensive coverage of all elements that compose plant life, only a rudimentary understanding of how plants alter biogeochemical cycling can be formed.

Recent decades have witnessed expanding interest regarding the role of lesser studied elements in plant nutrition, such as rare earth elements (REEs) and transition metals, driven by technological applications, environmental contamination concerns, and emerging evidence for biological functions.^4,5^ For instance, REEs, comprising Sc, Y, and the lanthanides, have shown beneficial and toxic effects on plant growth depending on their concentration, with beneficial mechanisms including membrane stabilization, enzyme activities, and chlorophyll interactions.^4,6^ Similarly, elements such as Ti, Si, and various heavy metals are increasingly recognized as beneficial nutrients at varying concentrations for their roles in stress tolerance, structural function, or as indicators of environmental exposure.^7,8^ By quantifying the distribution of less typically studied elements alongside essential elements in plant tissues and by partitioning these estimates into useful categories for modeling and research, a more inclusive picture of plant mineral nutrition may give rise to new insights regarding what elements play a role in plant nutrition.

Despite this growing recognition, global-scale data sets of plant elemental composition remain limited in scope and geographic coverage. The recently published NPKGRIDS provides comprehensive global data for N, P, and K fertilizer applications but does not address plant tissue concentrations or include trace elements.^9^ The StoichLife database represents a significant advance in global trait compilation, focusing on C, N, and P stoichiometry across plant and animal taxa, but similarly excludes most micronutrients and trace elements.^10^ Regional studies have documented major elemental patterns in specific ecosystems or crop systems,^11^ yet comprehensive analyses integrating multiple continents, soil types, and climate zones are largely absent from the literature.

Molar elemental ratios in plants serve as powerful diagnostic tools in ecological stoichiometry, plant physiology, and environmental assessment. The N:P ratio has emerged as a key indicator of nutrient limitation and ecosystem productivity,^12,13^ while K:Na ratios distinguish salt-tolerant from glycophytic species.^14^ Other ratios, such as Ca:Mg, Zn:Cd, and Si:Al, provide insights into soil adaptation, metal tolerance, and functional group classification.^1,8^ However, elemental ratios for REEs and many trace elements have not been systematically documented on a global scale, representing a significant knowledge gap in comparative plant biology and biogeochemical modeling.

The identification of hyperaccumulator species, or plants that concentrate metals to levels far exceeding normal plant tissue concentrations, has important applications in phytoremediation, biogeochemical prospecting, and understanding evolutionary adaptations to metalliferous soils.^15^ While hyperaccumulation thresholds have been established for several metals (Ni, Cu, Zn, Cd, Pb, Co, Cr, Mn, and As), systematic global inventories remain incomplete, and thresholds for many trace and REE have not been defined.^16^

The objectives of this study were to (1) compile and analyze a comprehensive global database of plant elemental composition spanning 52 elements across diverse taxa, environments, and geographic regions; (2) calculate global means and confidence intervals for plant tissue concentrations, with particular attention to elements lacking previous global estimates; (3) quantify elemental partitioning among plant organs and ecological groups; (4) compute key molar ratios and compare values to established literature ranges; (5) conduct phylogenetic analyses to assess evolutionary constraints on elemental composition; (6) apply multivariate approaches to identify compositional patterns distinguishing domesticated crops from wild species; and (7) systematically identify hyperaccumulator species based on established thresholds and evaluate taxonomic and geographic patterns in metal accumulation.

## Materials and Methods

### Data Collection

The literature was searched through Google Scholar for the following terms: “mineral nutrition”, “plant nutrition”, “plant elemental uptake”, “phytoremediation”, “plant elemental abundance”, along with similar terms. Only studies from peer-reviewed literature that reported quantitative plant tissue elemental concentrations together with plant identity and sampling location were selected for analysis. Only studies that reported values on a dry weight basis were used. Data from studies were composited into a collective database (available in supplemental information) by manually taking values from the studies, which included adjusting all values to part per million on a weight basis (i.e., mg kg^–1^). The following metadata were collected with each study: author name, URL, plant species, plant part sampled, edibility, latitude and longitude of study, photosynthetic system, life cycle, phylogentic information, and instrument type. Hydroponic and greenhouse studies that included additions of elements far exceeding environmental concentrations were not considered to maintain estimates of mineral grown plants. Because the database was assembled from published field studies spanning multiple years and regions, study dates were not sufficiently standardized to support formal temporal trend analyses across the compiled observations. Additionally, standardized soil data was seldom reported for studies and therefore was considered outside the scope of the meta-analysis.

### Spatial Analysis

Coordinate location data (i.e., latitude and longitude) were obtained from the studies included in the database. In cases where no coordinates were given, the coordinate values were extracted from stated locations in the paper. To standardize all values, coordinate data were converted to the World Geodetic System 1984 (WGS84). Coordinates were imported into ArcGIS Pro (version 3.5, Esri, Redlands, CA, USA) and then converted into point features, which were spatially joined to global data sets of soil pH and carbon,^17^ surface solar radiation from NASA POWER,^18^ Köppen climate classification,^19^ and soil groups.^20^ Because the study uses globally gridded soil products rather than site-specific soil analyses, the 26 soil groups and associated soil properties should be viewed as coarse descriptors of edaphic context as they cannot capture fine-scale variation in soil chemistry within individual sites.

### Distribution and Reliability of Collected Data

Smoothed density histograms were generated for the dry weight concentrations of each element. To assess how additional studies and information influenced the predictive accuracy for inference, cumulative mean learning curves (standardized and offset) for each element as meta-analytical effect sizes were plotted against the number of studies reporting each element. The learning curves reveal how much improvement can be expected from additional data and the point of diminishing returns, showing if the model is underfitting or overfitting as sample size changes. The learning curve code generates averages for each element by permuting (1000 times) the observed values and calculating the running (cumulative) mean as data were sequentially “accumulated”, identifying the expected mean trajectory and its 95% CI. The output standardizes and vertically offsets each element’s cumulative mean curve for clear multielement visualization, showing how average estimates stabilize with increasing sample size. In addition to learning curves, the entropy was assessed for each element to assess how uncertainty would be reduced with additional studies. The entropy code computes the cumulative empirical entropy (uncertainty) at each step as new values are added, repeatedly permuting the input and using histogram-based estimates for each subsample size, then summarizing the average, lower, and upper confidence bounds. The results are standardized and vertically offset to visually compare how entropy (data unpredictability or diversity) grows and stabilizes for each element as sample size increases.

### Global Estimates of Plant Tissues

Due to experimental differences between studies (e.g., environment, sampling bias, and instrumentation), the use of a mixed-effects model was evaluated to determine if using study as a random effect would improve model reliability. Akaike information criterion (AIC) and Bayesian information criterion (BIC) were used as model selection tools comparing the regressions of elemental concentration with study as a fixed effect or as a random effect. The AIC values were plotted for each element. Following the random effects model, global least-squares (LS) means were calculated for each element along with its 95% CI. Computation was performed using the *lme4* package^21^ in RStudio (v. 4.2). For comparison to raw measures of central tendency, mean and medians are reported in Table S1. For reporting purposes, concentrations < 0.01 mg kg^–1^ were reported as < 0.01 as these values approach detection limits of most instruments and may not be reliable across all studies considered. Molar ratios were calculated from global plant elemental means by using standard atomic weights. These ratios are intended as broad comparative indicators across functional groups and taxa rather than as site-specific measures of nutrient limitation or elemental balance.

### Accumulation Ratio

An accumulation ratio was computed for each element as the ratio of global plant concentration to a published global reference abundance,^35^ also included in Table S3. In the present study, the reference abundances correspond to continental crust values, as also indicated in Figure 3e, and are used here only as first-order benchmarks for broad biogeochemical comparison rather than as site-specific soil measurements for the individual plant samples. Because these reference values are coarse global averages, they do not capture local variation in parent material, pedogenesis, contamination, or management and therefore should not be interpreted as precise estimates of plant uptake relative to the soil chemistry at any specific sampling location.

**Figure 1:**
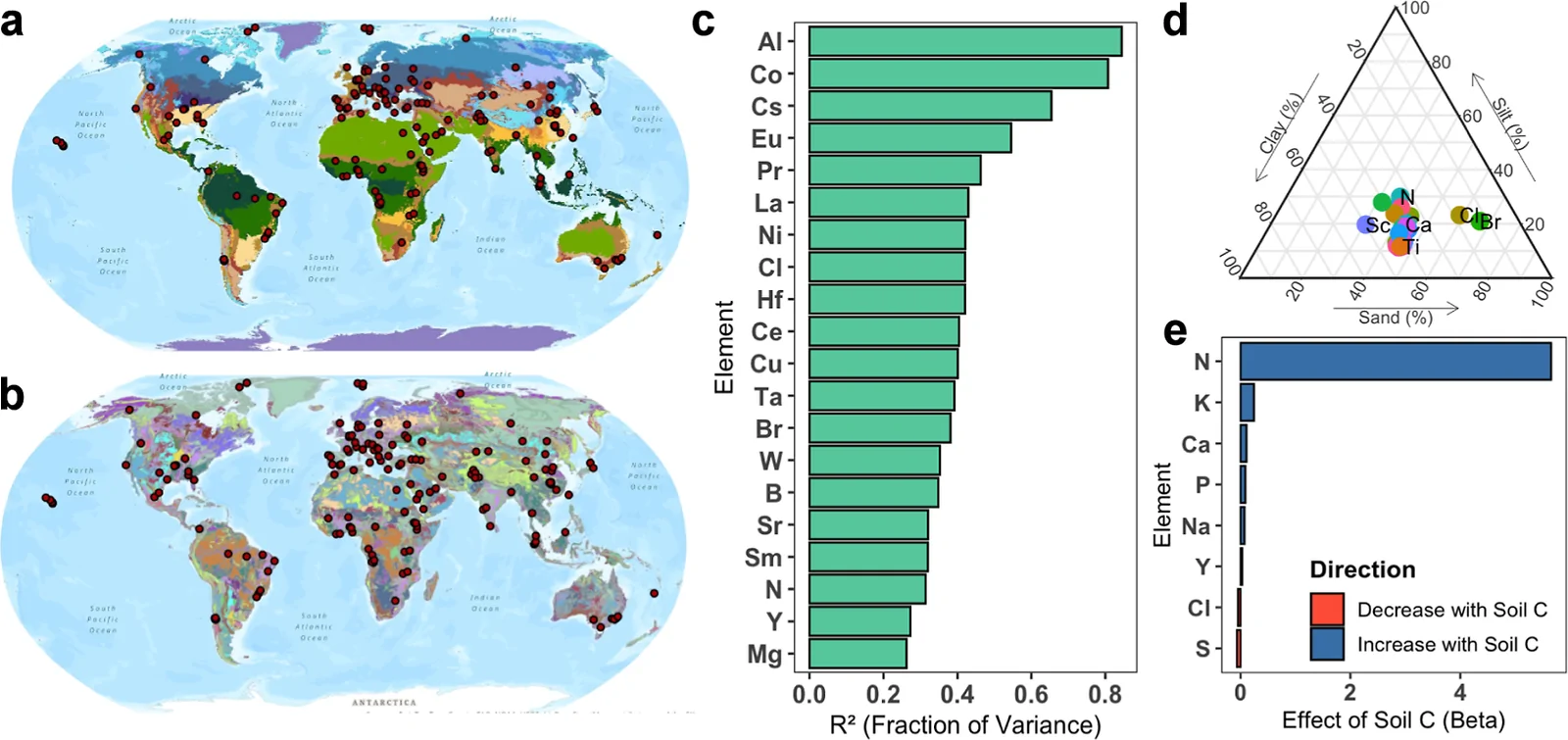
Global distribution of database studies by (a) Köppen climate classification and (b) soil orders. Colors in maps indicate discrete climates and soil orders. (c) Bar plot of the variance of plant tissue concentrations explained by soil order listed for elements with *R* ^2^ > 20%. The four elements with >50% variance were explained by high values in Acrisols. (d) Ternary diagram of soil texture overlaid with the geometric mean of each element. (e) Bar plot of the slope of each elemental linear model as an effect of soil C concentration showing only elements with a slope >|0.1|.

**Figure 2:**
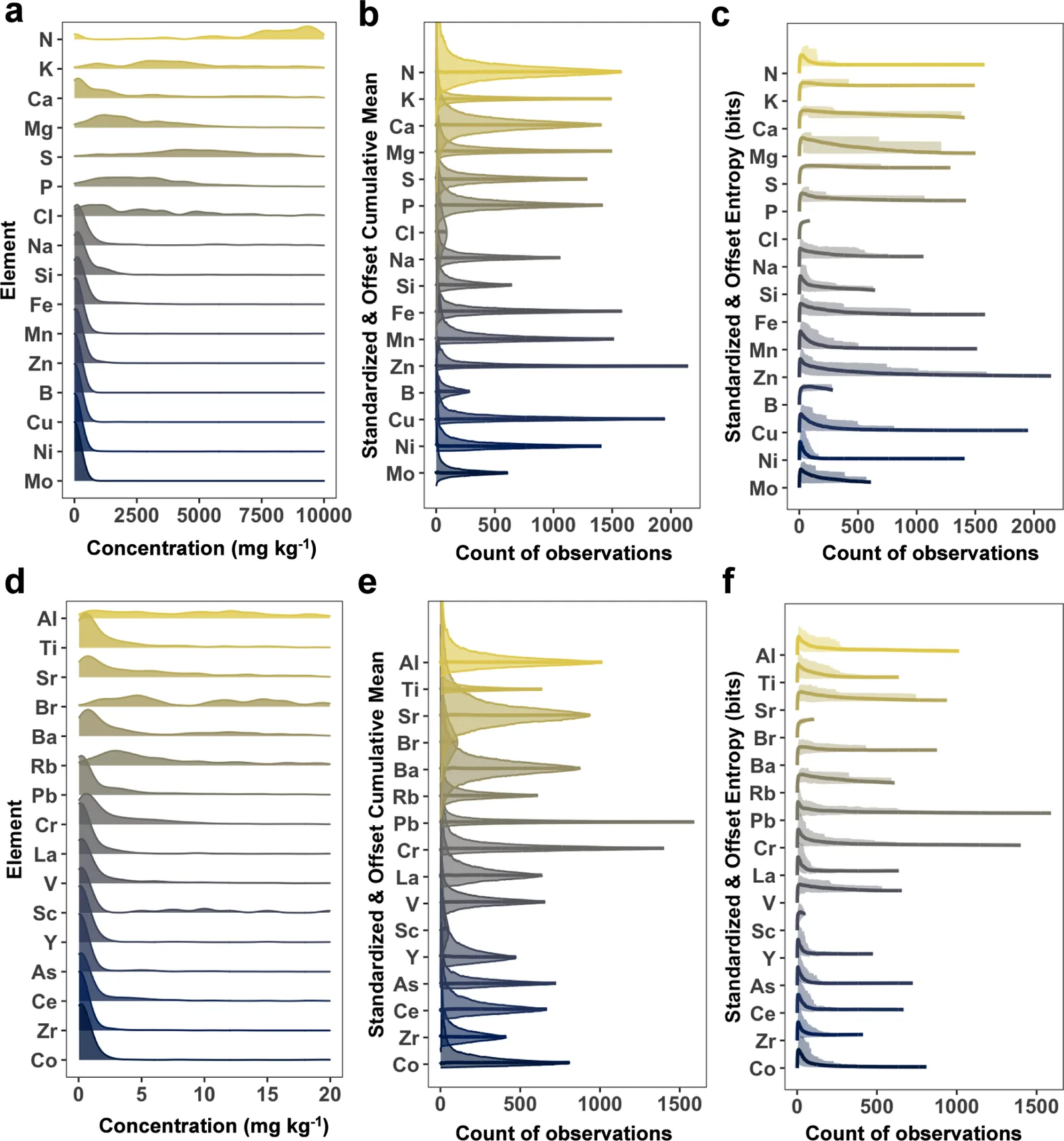
Meta-analytic summary statistics for (a–c) plant essential nutrients (i.e., N, K, Ca, Mg, S, P, Cl, Fe, Mn, Zn, B, Cu, Ni, and Mo) and common beneficial nutrients (i.e., Na and Si) and (d–f) most abundant nonessential elements on a dry weight basis for all data points in the database. (a,d) Density distributions of elemental concentrations (mg kg^–1^ dry mass) measured across global data sets ordered by abundance. (b,e) Cumulative mean learning curves (standardized and offset) for each element as meta-analytical effect sizes plotted against the number of studies reporting each element. (c,f) The shadowed area represents the 95% CI. Entropy measures of variability and uncertainty in elemental data sets.

**Figure 3:**
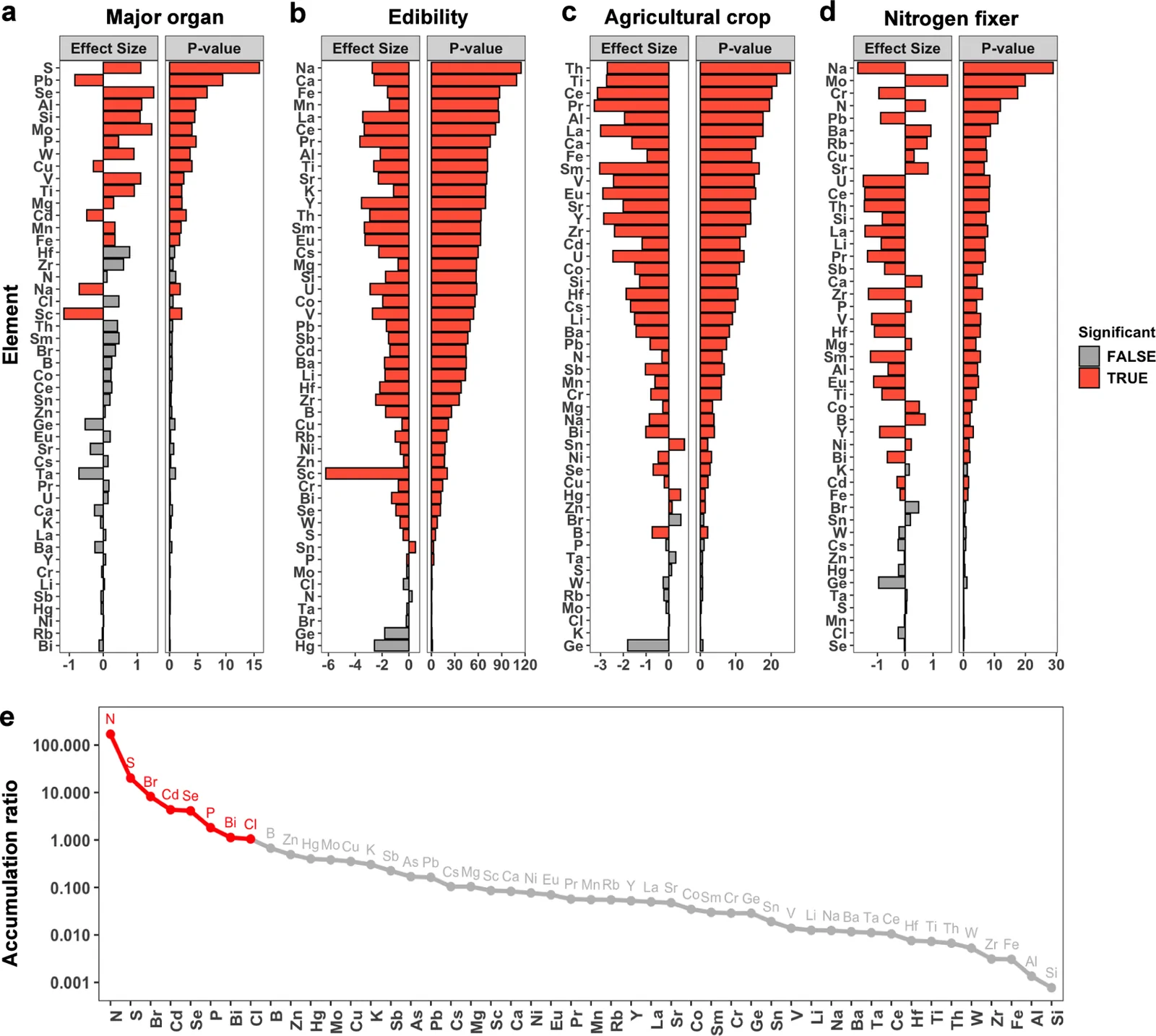
Abundance of elements in plant tissues as statistical tests and effect sizes of the elemental content of various plant forms (a–d) and accumulation ratio of each element (e). For elements in red (a–d), the *p*-value was significant (α < 0.05), while gray indicates a lack of significance. As binary indicators, the direction and length of the effect size bar indicates which binary is greater. The binary indicators are major organs (roots vs shoots) (a), edibility (no vs yes) (b), domesticated crop (no vs yes) (c), and nitrogen fixer (no vs yes) (d), reported as negative vs positive responses in the graphs, respectively. Accumulation ratio (molar) of the reported abundance of plant concentrations compared to the reported abundance of those elements in the continental crust of the Earth (e). An accumulation ratio of 1 indicates similar concentrations among plant and continental crust concentrations.

### Statistical Testing

Global estimates of plant elemental concentration were used to test four different hypotheses regarding the functionality of the plants: major organ (shoots or roots), edibility (yes or no), domesticated crop (yes or no), and symbiotic N-fixation ability (yes or no). Analysis of variance was performed for each of the four tests, and an effect size was generated to indicate the direction and magnitude of the effects. Global LS means were calculated for each element in its four different plant traits. Additionally, LS means were generated for each of the different plant types (trees, shrubs, vines, forbs, sedges, grasses, mosses, and ferns). Computation was performed using the *lme4* and *lmerTest* ^22^ packages.

### Phylogenetic Analysis

A plant phylogenetic tree was constructed matching species entries to a global megatree using the *V.PhyloMaker2* package,^23^ and then per-species elemental concentrations were calculated by fitting mixed effects models to extract species-level LS means (accounting for study as random effects). A phylogenetic signal was then quantified for each trait (Blomberg’s K and Pagel’s Lambda, which measures how much trait similarity is explained by phylogeny) using the *phytools* package.^24^ Three evolutionary models (Brownian Motion (BM), Ornstein–Uhlenbeck, and Early Burst (EB)) were fit to compare trait evolution mechanistically via likelihoods and information criteria, check for rate heterogeneity (branch-specific evolutionary rates), and reconstruct continuous ancestral states on the tree under the best-fit evolutionary model for each trait. Mathematically, the workflow combines permutation, linear mixed modeling, maximum likelihood model fitting, and comparative methods (variance partitioning, model selection via AIC, trait evolution simulation, and ML-based ancestral state estimation) to explore evolutionary patterns in large-scale multitrait data. The use of eXtreme Gradient Boosting (XGBoost) as un unsupervised ML tool was used for further analysis to assess the prediction ability of plant elemental concentrations to taxonomic coverage (phylum, class, order, family, and genus). The elements with the greatest contribution across taxonomic coverage were averaged and reported.

### Multivariate Analysis

Multivariate analyses were performed on the entire database using JMP Pro (version 17.2.0). The projections consisted of (a) uniform manifold approximation and projection (UMAP), (b) t-distributed stochastic neighbor embedding (t-SNE), (c) principal coordinate analysis (PCA), and (d) the contribution of variables to principal coordinates.

### Hyperaccumulator Identification

Hyperaccumulators are defined as species with concentrations equal to or greater than the element-specific threshold in the indicated organ, based on previously established concentrations.^15^

### Data and Code Availability

The database generated from this study is available as a spreadsheet in the Supporting Information. The database and all R code used to generate the analyses are available at https://github.com/MoonVillageAssociation/LAST-Plant_Nutrition.

## Results and Discussion

### Geographical Distribution of Data

The collected data points, encompassing 21 Köppen climate classes across 6 continents, 73 countries, and 26 soil groups, produced a data set with substantial representation of the world’s bioclimatic and edaphic variability (Table 1; Figure 1ab). Soil pH values of the analyzed locations ranged from 4.7 to 9.0. Solar radiation (All Sky Insolation Clearness Index) ranged from 0.27 to 0.74 and soil C from 0 to 209 g kg^–1^. When assessing the influence of soil order on plant elemental concentrations, the variance for Al, Co, Cs, and Eu was >50% and was found to be associated with elevated plant concentrations in ultisols (Figure 1c). Ultisols are highly weathered soils in tropical areas with acidic pH that likely drive the greater uptake of these metals compared to other soil orders. Soil texture was well represented across the database with only the halogens Cl and Br having greater prevalence in sandy soils, although these elements had a lower number of data points in the study and may be influenced by study conditions (Figure 1d). As for the elemental content of soils, the only meaningful interpretations provided by soil concentrations originating from the geospatial data were that of soil C increasing N uptake in plants, with each one percent increase in soil C contributing to approximately 5 mg kg^–1^ more N in the plant (Figure 1e). In general, C and N cycles are intimately linked via redox reactions and their cycling through plants. Broad database coverage enables the detection of large-scale trends and regional adaptations, strengthens the reliability and generalizability of findings, and helps avoid biases that can arise from over-representing certain regions.

**Table 1:** Database Coverage of Climate Classes and Soil Orders^a^

climate class	count	soil order	count
tropical, rainforest	7	alfisols	29
tropical, monsoon	6	andisols	1
tropical, savannah	17	aridisol	2
arid, desert, hot	17	aridisols	11
arid, desert, cold	2	entisols	25
arid, steppe, hot	5	histosols	1
arid, steppe, cold	15	Inceptisols	11
temperate, dry summer, hot summer	19	Mollisols	12
temperate, dry summer, warm summer	13	oxisols	9
temperate, dry winter, hot summer	4	Spodosols	6
temperate, dry winter, warm summer	4	Ultisols	11
temperate, no dry season, hot summer	43	Vertisols	8
temperate, no dry season, warm summer	23	not mapped	30
cold, dry summer, hot summer	1		
cold, dry summer, warm summer	1		
cold, dry summer, cold summer	1		
cold, dry winter, hot summer	2		
cold, dry winter, warm summer	1		
cold, dry winter, cold summer	2		
cold, no dry season, hot summer	3		
cold, no dry season, warm summer	11		
cold, no dry season, cold summer	2		
polar, tundra	7		

a Counts represent the number of studies represented by each category.

Although the database spans broad climatic and edaphic gradients, it should be interpreted primarily as a spatial synthesis rather than a temporally resolved record. Many of the underlying studies were conducted at different times and with limited temporal replication, which prevents direct inference about long-term trends in plant elemental uptake under warming, altered rainfall regimes, or changing hydrological conditions.

Spatially comprehensive data sets are necessary for discerning biogeochemical patterns; yet, most published plant nutrient surveys have focused on regional or biome-specific sampling or individual crop systems.^25^ Obtaining global estimates for the elemental composition of plants will be affected by local and regional conditions, with plant age, stressors, and climate^26^ having effects on plant uptake. Therefore, it is necessary to encompass more than a few climate zones or soil orders and integrate multicontinent, soil carbon, and radiation gradients to produce robust averages.

### Statistical Distribution and Reliability of Data

Assessing the statistical distribution and reliability of elemental concentration data is fundamental to understanding the robustness and potential uncertainties of global plant nutrition patterns. Elemental concentration distributions for soil-derived plant essential nutrients (Figure 2a) are characteristically right-skewed, with a large majority of observations clustered at relatively low concentrations and a small subset of samples exhibiting extremely high values. Macronutrients (i.e., N, K, P, S, Mg, and Ca) and Cl display not only the highest overall concentrations but also the widest distribution. For these elements, cumulative mean values stabilize rapidly with an increasing study count (Figure 2b), indicating robust and consistent global estimates. Entropy analysis (Figure 2c) quantifies uncertainty in the elemental concentration distributions. For well-studied elements (N, K, P, and Ca), entropy quickly declines with the accumulation of studies and stabilizes at relatively low values, again indicating reliable and predictable global patterns. Conversely, micronutrients exhibit higher entropy even at larger study numbers, suggesting persistent knowledge gaps and higher variability in the current data coverage.

Extending to nonessential elements (Figure 2d), right-skewed distributions persist, but concentrations are orders of magnitude lower, with most values below 20 mg kg^–1^. Micronutrients (i.e., Fe, Zn, Mn, B, Cu, Ni, Mo, and Cl), such as Mo and Ni, that are less studied show pronounced fluctuations in cumulative means, reflecting less certainty in their global averages. Cumulative mean trajectories (Figure 2e) are highly unstable at lower study counts and often remain variable even at higher sampling densities, indicating both a lack of coverage and substantial heterogeneity in how these elements accumulate across global plant lineages and environments. Entropy values for nonessential elements (Figure 2f) are high and show only modest declines with increased sampling, supporting the conclusion that plant uptake of these less-studied elements remains more uncertain and variable at the global scale.

While not reflected in the current database, the chemical speciation of elements critically determines their bioavailability and uptake by plants through distinct transport pathways and mechanisms. For example, iron (Fe) uptake exemplifies this principle; in nongraminaceous plants, Fe^3+^ must be reduced to Fe^2+^ at the root surface by the ferric chelate reductase FRO2 ^29^ before uptake via the high-affinity transporter IRT1,^30^ whereas graminaceous species secrete phytosiderophores that chelate Fe^3+^ for acquisition as complexes through the YS1/YSL transporters.^31^ The Fe example showcases that elemental speciation fundamentally governs transport mechanisms, cellular compartmentalization, and bioavailability in plants, thereby controlling both nutrient acquisition and the accumulation of elements. The large sample sizes and reliability of the generated data allow for the estimation of plant uptake on a global scale while overriding such specific issues with speciation.

### Summary Statistics

Across all elements, excluding Sn and Mo, there were significant differences between mixed effects vs fixed effects models. As the mixed-effects models utilize study as a random intercept, variance due to underlying biological or study factors (e.g., environment, sampling bias, and instrumentation) drove very extreme significance for Zn, Pb, P, N, and Cd. Improvements remained highly significant for all elements, except Sn (*n* = 582) and Mo (*n* = 611). Comparing AICs for all elements (Figure S1), the support for improved global mean calculation in mixed-effects models was demonstrated by lower AIC for all elements. Thus, LS means were computed for each element.

### Global Means of Plant Tissues

The globally computed abundance of elements in plant tissues was estimated by using LS means for each element (Table 2). Elemental concentrations for macronutrients and micronutrients are consistent with standard reference works including crops and wild species.^28,32^ However, there have been no prior global mean estimates for plant tissue concentrations of REEs (La, Ce, Sm, Eu, Pr, and Y), alkali metals not usually considered essential (Cs and Rb), minor and trace transition metals (Zr, Hf, Ta, and W), post-transition and heavy metals (Bi, Th, and U), and metalloids (As and Ge). Many nonessential elements exist in plant tissues in higher concentrations than micronutrients, especially Mo; in decreasing order, these include Na, Si, Al, Ti, Sr, Br, Ba, Rb, Pb, Cr, La, Nd, V, Sc, Y, As, Ce, Zr, Co, and Cs. While global estimates of plant concentrations of REEs and trace elements are a novel finding, these elements tend to have relatively large uncertainty and variation in their estimates.

**Table 2:** Global Means of 52 Element Concentrations in Plant Dry Weight Tissues and Their 95% Confidence Intervals^a^

element	global mean	confidence interval (95%)	observations (*n*)
	mg kg^–1^ plant dry weight		
N	14,059	6,413–30,819	1,580
K	7,075	4,972–10,068	1,497
Ca	2,124	1,127–4,004	1,410
Mg	1,556	940–2,576	1,502
S	1,253	516–3,042	1,288
P	1,188	692–2,038	1,420
Cl	388	16–9,115	91
Na	300	150–598	1,056
Si	240	35–1,641	646
Fe	139	88–220	1,586
Al	110	42–288	1,015
Mn	42.9	27-68	1,516
Zn	33	23–48	2,146
Ti	27.9	5.2–150	638
Sr	15.2	6–39	939
Br	13.2	4.7-37	107
B	11.4	6.1–21	286
Cu	9.89	7.4–13	1,950
Ba	7.31	1.7–32	876
Rb	4.61	2.8–7.8	611
Ni	3.59	2.0–6.3	1,409
Pb	2.78	1.5-5.4	1,617
Cr	2.64	1.4-4.9	1,401
La	1.54	0.20–12	638
Nd	1.37	0.10–18	533
V	1.34	0.49–3.6	656
Sc	1.2	0.02–67	52
Y	1.1	0.01–101	475
As	0.81	0.16–4.1	725
Ce	0.66	0.06–7.1	667
Zr	0.6	0.06–5.9	413
Co	0.6	0.19–1.9	811
Cs	0.51	0.02–16	599
Mo	0.42	0.32–0.54	611
Pr	0.4	0.01–12.9	582
Cd	0.39	0.21–0.75	1,357
Se	0.37	0.15–0.91	741
Li	0.3	0.09–1.1	740
Bi	0.18	<0.01–20	311
Sm	0.14	0.01–1.6	479
U	0.12	0.01–2.1	592
Sb	0.09	0.03–0.28	635
Th	0.07	0.02–0.35	545
Eu	0.07	<0.01–1.2	505
Yb	0.07	<0.01–1.0	479
Sn	0.04	0.02–0.10	582
Ge	0.04	0.01–0.24	26
Hf	0.04	<0.01–1.6	365
Tb	0.02	<0.01–0.55	479
Hg	0.02	<0.01–0.07	260
W	0.01	<0.01–0.11	536
Ta	0.01	<0.01–0.06	276

a Global means were computed as LS means using study as the random effect.

### Elemental Distribution in Various Functional Categories and Plant Types

Partitioning plant functional categories into four binary indicators (major organs, edibility, domestication, and N fixation ability) reveals many significant effects and large effect sizes. Because developmental stage and cultivar information were inconsistently reported, the organ-level and functional-group comparisons represent mean conditions across a range of growth stages and varieties and should be interpreted as macro-scale patterns rather than precise developmental trajectories. Major organ identity (roots vs shoots) reveals that Pb, Sc, Na, Cd, and Cu concentrations are increased in roots, whereas all other significantly different elements are elevated in shoots (Figure 3a and Table 3). Concentrations of nearly all elements were increased in nonedible and wild plants compared to edible and domesticated plants, except for elevated Sn in edible plants and Sn and Hg in domesticated plants (Figure 3b,c and Table 2). Furthermore, because humans consume mineral-grown plants, human exposure to lesser-studied elements through plant consumption may have considerable interest to human nutrition as well. Concentrations of elements between N fixers and non-N fixers revealed variable preferences for Mo and Na, with N fixers taking up less nonessential nutrients (i.e., Na, Cr, Pb, U, Ce, Th, Si, La, Li, Pr, Sb, Zr, V, Hf, Sm, Al, Eu, Ti, Y, Bi, and Cd) and more essential nutrients (i.e., Mo, N, Cu, Ca, P, Mg, Co, B, and Ni) and only a few nonessential nutrients (i.e., Ba, Rb, and Sr) (Figure 3d and Table 2).

**Table 3:** Global Means (mg kg^–1^ Plant Dry Weight) of Plant Nutrition by Functional Category (Major Organ, Edibility, Domesticated Crop, and N Fixer)^a^

element	major organ		edibility		domesticated crop		nitrogen-fixer	
	roots	shoots	no	yes	no	yes	no	yes
Al	39	120	371	44	190	27	114	61
As	0.89	0.79	0.83	0.08	0.94	0.24	0.91	0.34
B	9.2	12	33	5.8	16	7.6	11	22
Ba	9.0	7.0	46.2	7.4	14.3	3.4	6.7	17
Bi	0.20	0.17	0.06	0.02	0.28	0.10	0.20	0.11
Br	10	14	16	13	11	18	13	20
Ca	2,705	2,088	10,589	782	3,232	638	2,034	3,682
Cd	0.62	0.38	0.74	0.18	0.58	0.18	0.40	0.30
Ce	0.54	0.69	3.56	0.13	2.10	0.09	0.89	0.21
Cl	257	406	527	349	384	395	395	307
Co	0.49	0.62	1.69	0.24	0.95	0.21	0.58	0.95
Cr	2.7	2.6	3.8	1.7	3.3	1.5	2.8	1.1
Cs	0.47	0.54	2.65	0.28	0.98	0.18	0.52	0.40
Cu	13	9.6	12	7.1	10	8.6	9.7	13
Eu	0.06	0.07	0.35	0.01	0.11	0.01	0.07	0.02
Fe	102	144	398	81	182	70	141	117
Ge	0.06	0.03	0.10	0.02	0.10	0.02	0.05	0.02
Hf	0.02	0.05	0.28	0.03	0.07	0.01	0.04	0.01
Hg	0.02	0.02	0.09	0.01	0.02	0.03	0.02	0.01
K	7,600	7,011	16,105	5,099	7,112	6,957	7,002	8,075
La	1.5	1.6	6.8	0.22	2.3	0.12	1.6	0.38
Li	0.29	0.30	0.87	0.15	0.55	0.12	0.33	0.14
Mg	1,165	1,578	2,620	1,165	1,663	1,272	1,526	1,904
Mn	32	44	103	24	51	28	43	42
Mo	0.12	0.49	0.48	0.41	0.44	0.39	0.37	1.7
N	12,713	14,181	12,851	16,159	14,983	11,058	13,490	27,871
Na	584	287	2319	150	375	160	355	64
Nd	0.99	1.5	4.8	0.14	3.9	0.16	1.4	0.37
Ni	3.6	3.6	4.4	2.3	4.1	2.6	3.5	4.4
P	778	1,220	1,645	1,393	1,234	1,089	1,174	1,457
Pb	5.9	2.5	5.4	1.0	3.4	1.5	3.0	1.2
Pr	0.35	0.42	1.93	0.05	1.96	0.07	0.60	0.15
Rb	4.7	4.6	9.8	3.5	4.9	4.0	4.4	9.6
S	427	1,292	1,582	1,027	1,201	1,357	1,251	1,308
Sb	0.09	0.08	0.12	0.03	0.09	0.03	0.09	0.04
Sc	2.6	0.80	-	0.14	-	-	-	-
Se	0.09	0.42	0.77	0.29	0.48	0.24	0.37	0.36
Si	85	250	518	90	358	99	248	108
Sm	0.10	0.16	1.09	0.04	0.25	0.01	0.15	0.04
Sn	0.04	0.04	0.03	0.05	0.03	0.06	0.04	0.05
Sr	21	14	70	7	35	5	14	32
Ta	0.01	0.01	0.01	0.01	0.01	0.01	0.01	0.01
Tb	0.01	0.02	0.12	<0.01	0.04	<0.01	0.02	0.01
Th	0.06	0.08	0.54	0.03	0.11	0.01	0.08	0.02
Ti	13	33	101	7	49	3	29	12
U	0.11	0.13	0.64	0.04	0.32	0.03	0.13	0.03
V	0.51	1.52	3.89	0.26	2.16	0.19	1.41	0.42
W	<0.01	0.01	0.02	0.01	0.01	0.01	0.01	0.01
Y	1.0	1.1	2.5	0.07	2.6	0.15	1.2	0.46
Yb	0.04	0.07	0.50	0.02	0.11	0.01	0.07	0.02
Zn	31	33	47	31	32	37	33	32
Zr	0.38	0.69	3.74	0.32	0.97	0.09	0.63	0.17

a Global means were computed as LS means using study as a random effect.

The accumulation ratios of each element (global plant concentration ÷ global soil concentration^35^) reveal that the macronutrients N, S, and P exhibit accumulation ratios greater than 1 with N having an order of magnitude larger ratio than all other elements (Figure 3e). The nonmetals Br, Cl, and Se; the transition metal Cd; and post-transition metal Bi were the only elements to have positive accumulation ratios. All other elements contained negative ratios, with Fe, Al, and Si being the lowest three elements of all which correspond to their high abundance in the terrestrial crust. These ratios should be interpreted as global benchmarks rather than site-level soil measurements; accordingly, they are most useful for broad comparative context and not for inferring local plant–soil enrichment factors for individual records.

The partitioning of metals and nutrients among roots and shoots is substantiated by physiological literature and meta-analyses reporting enhanced root uptake and sequestration of elements such as Pb, Cd, and Cu in both hyperaccumulators and normal plants.^1^ Increased concentrations of Na, Ca, Fe, Mn, La, and Ce in nonedible plant parts represent lower loading of metals into edible plants or edible plant parts. Reduced concentrations of metals such as Th, Ti, Ce, Pr, Al, and La in domesticated crops reflect breeding efforts to reduce metal accumulation and enhance nutrient density of modern agronomic varieties.^25^ Higher Mo and N concentrations in N-fixers are consistent with the role of Mo as a cofactor in nitrogenase, the enzyme underlying symbiotic N fixation, but the broader elemental pattern likely also reflects habitat filtering, species composition, and differences in ion exclusion rather than a generalized increase in nutrient uptake across all elements. Accumulation ratios comparing plant tissues to crustal abundance are only sporadically reported in prior literature, mainly for select macronutrients and contaminants; the present comprehensive ratios for less-studied elements provide new context for evaluating biogeochemical cycling. Because the compiled studies do not consistently report total or plant-available soil micronutrient stocks, land-use history, or duration of cultivation, the present analysis cannot directly determine whether long-term agriculture has depleted or replenished micronutrient reserves relative to forests, grasslands, wetlands, or other native systems.

Elemental concentrations across plant types (Table 4 and Figure 4a) reveal distinct patterns of elemental accumulation that reflect physiological and ecological differences among plant functional groups. Ferns exhibit an enrichment of REEs such as Pr, Sm, Tb, Nd, and Yb, whereas vines are enriched in heavy metals and actinides, including U, Th, La, and V. Mosses display high accumulations of Si and Ti but show depletion in certain heavy metals and REEs. Sedges are characterized by elevated levels of Mn and selected REEs. Grasses tend to have lower concentrations of many elements, which may reflect different nutrient requirements or uptake capabilities. Trees and shrubs both maintain moderate elemental concentrations with trees notably enriched in Li, Na, and Se, likely reflecting their structural and physiological demands. Forbs generally cluster near median elemental concentrations but show increased levels of Mo. Hierarchical clustering of the plant types shows that trees and vines cluster tightly, associated with shrubs. Sedges and forbs cluster tightly, which is associated with grasses. Mosses and ferns are highly separated from the other plant types (Figure 4b).

**Table 4:** Global Means (mg kg^–1^ Plant Dry Weight) of Plant Nutrition by Plant Type (Fern, Forb, Grass, Moss, Sedge, Shrub, Tree, and Vine)^a^

element	fern	forb	grass	moss	sedge	shrub	tree	vine
Al	-	73	40	154	-	87	59	273
As	-	2.0	0.96	0.16	2.0	2.7	2.5	6.8
B	-	21	1.7	5.4	-	12	12	24
Ba	-	9.7	0.77	28	-	13	11	32
Bi	-	0.36	0.32	0.01	-	0.55	0.26	0.71
Br	45	14	11	23	-	39	4.8	-
Ca	1,120	3,992	282	538	3,378	6,839	6,037	8,649
Cd	0.43	0.96	0.30	0.09	1.8	0.56	0.43	0.77
Ce	5.3	0.33	0.10	0.83	1.5	0.43	0.21	2.1
Cl	1,244	446	413	844	-	-	168	-
Co	-	0.87	0.17	0.31	-	0.57	0.39	1.4
Cr	-	2.3	4.1	2.0	2.1	1.8	2.5	4.1
Cs	-	1.1	0.18	0.22	-	1.2	0.98	2.7
Cu	10	14	6.3	6.0	5.3	13	12	17
Eu	1.5	0.06	0.02	0.01	0.52	0.150	0.07	0.3
Fe	163	173	80	105	54	114	97	275
Ge	-	0.01	0.02	0.10	-	-	-	-
Hf	-	0.02	0.02	0.11	-	0.03	0.03	0.08
Hg	0.02	0.02	0.01	0.03	0.01	0.03	0.01	0.03
K	9,077	8,963	2,493	9,115	-	6,598	7,402	12,515
La	40	1.7	0.44	0.41	12	3.0	1.4	12
Li	-	0.29	0.10	-	-	0.43	1.3	1.3
Mg	1,064	2,289	979	708	-	1,985	2,067	2,986
Mn	12	51	20	36	272	59	36	67
Mo	-	0.79	0.29	0.31	-	0.25	0.14	0.47
N	26,807	10,192	11,202	31,606	8,292	22,893	16,735	17,038
Na	914	288	220	127	-	390	867	1,604
Nd	35	0.78	0.31	0.43	9.3	2.5	1.1	5.1
Ni	-	5.1	3.5	1.3	7.3	6.0	6.1	7.3
P	1,270	1,330	1,095	1,554	1,281	1,080	862	1,562
Pb	3.6	3.9	1.7	1.4	1.8	3.4	2.4	7.3
Pr	14	0.18	0.07	0.17	3.1	0.67	0.27	1.3
Rb	-	10	1.6	3.7	-	8.6	6.0	12
S	-	1,394	1,112	1,890	1,221	707	1,213	1,909
Sb	-	0.08	0.03	0.14	-	0.08	0.10	0.13
Sc	187	0.22	0.14	0.13	49	22	-	-
Se	-	0.40	0.31	0.13	-	0.30	0.74	0.38
Si	-	170	110	3378	-	192	201	389
Sm	4.4	0.13	0.05	0.06	-	0.32	0.16	0.68
Sn	-	0.04	0.02	0.11	-	0.03	0.05	0.03
Sr	-	16	1.1	40	-	24	23	37
Ta	-	0.01	0.01	0.01	-	0.01	0.01	0.01
Tb	0.47	0.02	0.01	0.01	-	0.04	0.02	0.09
Th	-	0.05	0.02	0.14	-	0.04	0.03	0.27
Ti	-	5.7	1.7	93	-	4.4	3.5	15
U	-	0.19	0.07	0.09	-	0.14	0.16	1.0
V	-	1.5	0.72	1.2	-	1.0	1.2	5.4
W	-	0.00	0.00	0.11	-	0.00	0.00	0.00
Y	16	0.60	0.17	0.42	4.3	1.7	0.82	3.7
Yb	1.7	0.07	0.03	0.02	-	0.17	0.09	0.4
Zn	35	39	33	36	38	32	22	36
Zr	-	0.49	0.37	0.72	-	0.53	0.69	2.0

a Global means were computed as LS means using study as a random effect.

**Figure 4:**
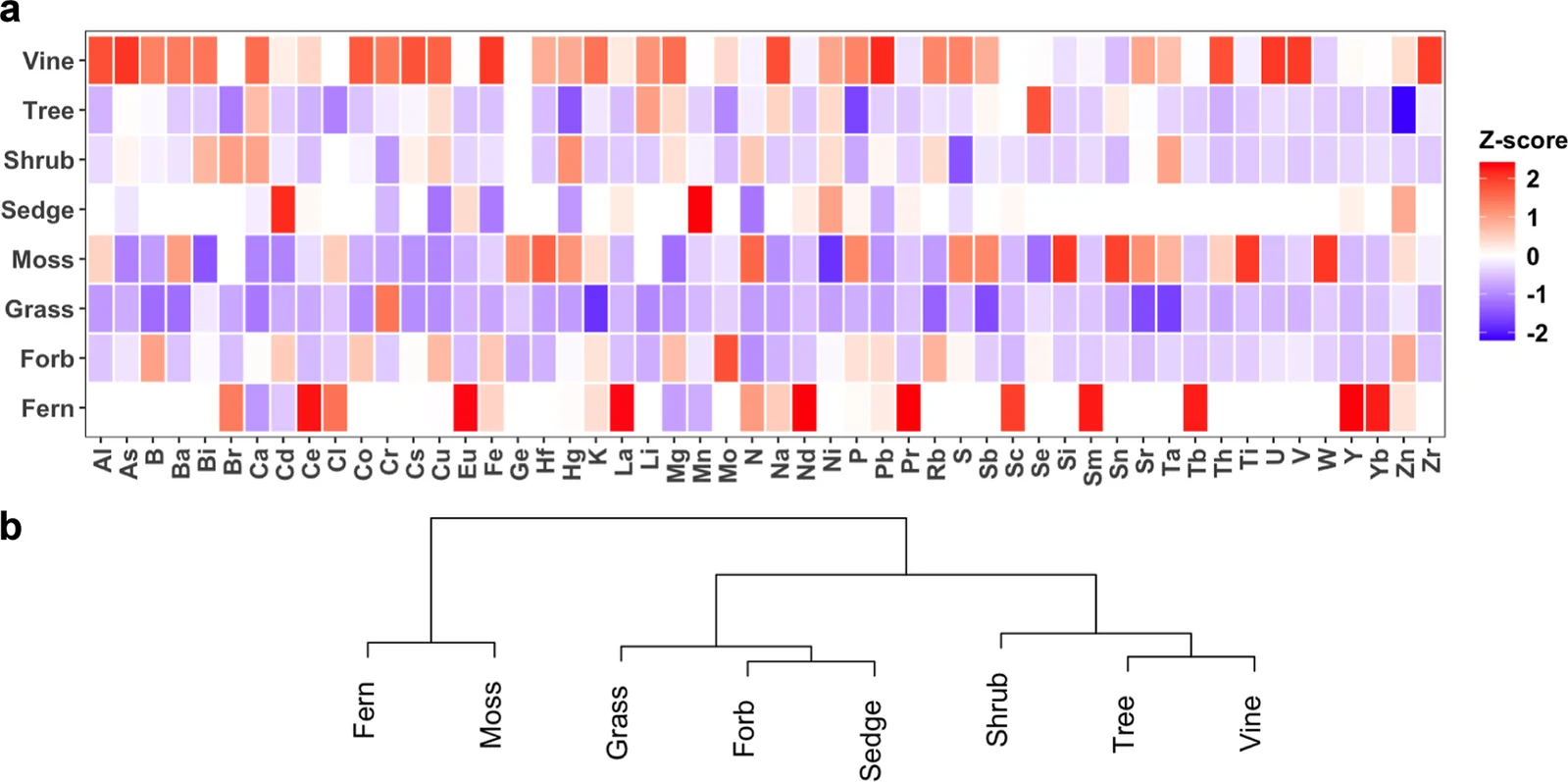
Heatmap of elemental concentrations (*z*-score normalized for each element) in various plant types (a). Hierarchal clustering of the various plant types (b).

### Phylogenetic Analysis

Global-scale analyses of plant elemental composition reveal that nutrient traits are far from being phylogenetically random. The phylogenetic framework and taxonomic coverage displayed in the circular dendrogram (Figure 5a) encompass all major terrestrial plant lineages, including angiosperms, gymnosperms, pteridophytes, and bryophytes, providing a high-resolution scaffold for comparative trait analysis. The distribution of tip colors across the tree confirms the greatest taxonomic representation of angiosperms, followed by bryophytes, and to a lesser extent, gymnosperms and pteridophytes.

**Figure 5:**
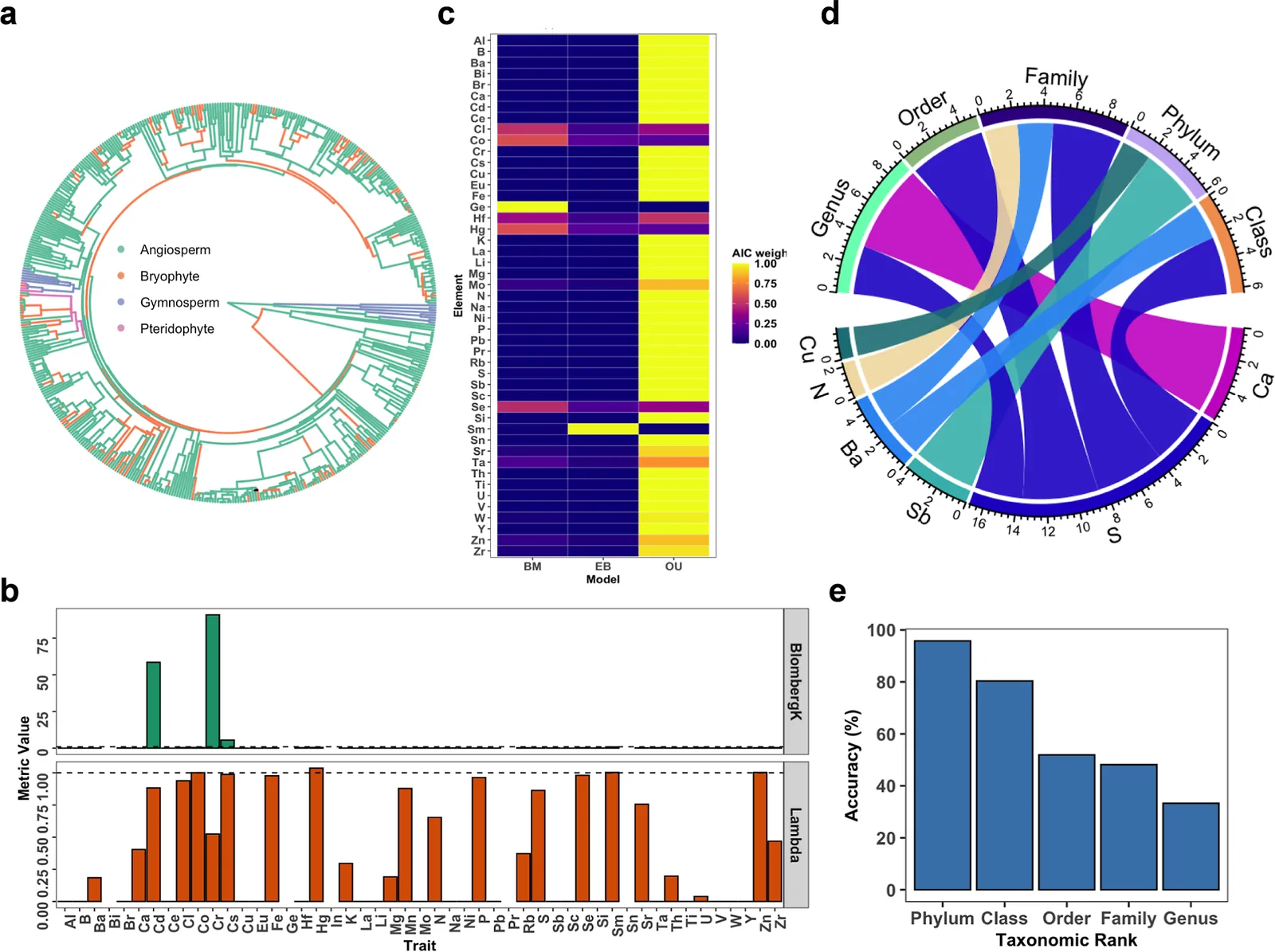
Phylogenetic and evolutionary analysis of plant elemental concentrations across major terrestrial lineages. (a) Circular phylogeny depicting sampled taxa with tip colors denoting major plant groups: angiosperms (teal), bryophytes (orange), gymnosperms (purple), and pteridophytes (pink), illustrating broad taxonomic and evolutionary coverage. (b) Phylogenetic signal for each element measured by Blomberg’s *K* (top; *y*-axis) and Pagel’s λ (bottom; *y*-axis). Blomberg’s *K* values >1 indicate stronger than expected phylogenetic clustering; Pagel’s λ values approaching 1 reflect high lineage-level dependence. (c) Model selection results for 34 elements, showing normalized AIC weights for three evolutionary models: BM, EB, and Ornstein–Uhlenbeck (OU) fit to each element. AIC weight near 1 (yellow) indicates strongest support. (d) Chord diagram of the top 14 elements (based on feature importance gain) for predicting taxonomic classification based on unsupervised machine learning (xgboost). The width of element ribbons indicates the mean feature importance for that element across the taxonomic groups. (e) Model accuracy of xgboost across taxonomic coverage.

Evolutionary model selection highlights stabilizing selection through element-wise Akaike weightings for three canonical models of continuous trait evolution (Figure 5b). The models used were BM, a random walk with no directional bias; EB, a rapid initial divergence followed by decelerating evolution; and Ornstein–Uhlenbeck (OU), a mean-reverting process reflecting stabilizing selection toward an optimum. Bright-yellow cells along the OU column (AIC weight ≥0.90) dominate the heatmap, whereas BM and EB receive negligible support for most elements. The OU dominance indicates that elemental concentrations are typically best explained by stabilizing selection (OU), rather than unconstrained drift (BM) or adaptive radiations confined to early lineage splits (EB). Exceptions to the OU dominance were Cl, Co, Ge, Hg, Se, and Sm, which were <1 mg kg^–1^ across plant tissues, except for Cl. OU dominance suggests that physiological or environmental optima constrain nutrient uptake and storage across evolutionary time with deviations from these optima rapidly corrected by selective pressure. The pattern is especially pronounced for macronutrients (i.e., N, P, and K) but extends to several micronutrients (i.e., Zn and Mn), signaling widespread evolutionary conservatism in plant stoichiometry.

Quantifying a phylogenetic signal using Blomberg’s K and Pagel’s λ deploys two complementary statistics to measure phylogenetic dependence. Bloomberg’s *K* with values >1 indicate stronger similarity among close relatives than predicted by BM; *K* < 1 denotes trait lability, whereas in Pagel’s metric λ ≈ 1 signals strong phylogenetic dependence and λ ≈ 0 implies independence. In Blomberg’s K, Cd and Cr surpass the *K* = 1 benchmark, denoting extreme phylogenetic clustering (Figure 5c green bars). The remaining elements register low values (*K* ≈ 0.3–0.9), consistent with moderate but significant lineage dependence. In Pagel’s λ, many elements yield λ > 0.75, with several approaching unity (Figure 5c red bars). These high values corroborate the OU findings, indicating that closely related species exhibit strongly correlated elemental profiles. Collectively, the dual-metric evidence confirms that evolutionary history is a primary determinant of plant elemental composition, aligning with theoretical expectations that slower evolutionary rates or strong stabilizing forces elevate phylogenetic signals.

The convergence of high OU support, elevated λ, and above-neutral *K* values provides evidence that plant nutrient traits are under pervasive stabilizing selection, gravitating toward lineage-specific optima rather than diffusing freely through trait space. The phylogeny highlights coherent nutrient signatures within major clades, suggesting that shared physiological architectures or habitat affinities impose similar elemental demands and uptake capacities across related taxa.

While macronutrients universally exhibit strong constraints, certain trace elements display mixed model support and lower signal metrics, implying either recent adaptive shifts or greater ecological plasticity in their acquisition and storage. Concordance between likelihood-based model selection and independent signal metrics strengthens the confidence in the inferred evolutionary regime.

The XGBoost prediction of taxonomic classification (phylum, class, order, family, and genus) by plant elemental values followed that the most important elements for predicting taxonomy were S > Ca > N > Zn > Ba > Cu > Pb > Sb > Cd > P with minor prediction gain thereafter (Figure 5d). Across all levels, S represented the highest predictor of taxonomy, with other elements having particular importance to specific groups. For example, genus was highly predicted by Ca, family by Ba and N, and phylum by Sb and Cu. Accuracies across all taxonomic coverages ranged from 95.8 to 33.3% (Figure 5e).

### Dimensionality-Reduction and Multivariate Analysis

Dimensionality reduction and multivariate analyses reveal distinct elemental profiles for cultivated vs wild plants. The t-SNE (Figure 6a) and UMAP (Figure 6b) projections converge on similar outcomes. Cultivated samples (red; *n* = 1657) occupy less cohesive and more diffuse regions, whereas wild taxa (blue; *n* = 2852) form a higher-density overlapping cloud. The broader scatter among cultivated plants reflects directed evolution and human-mediated nutrient management. In contrast, the tight clustering of wild plants shows convergent element stoichiometry driven by greater ecological and phylogenetic heterogeneity. Separation along both nonlinear embeddings is most pronounced along the first axis, indicating that a common combination of elements rather than a single nutrient underlies the divergence.

**Figure 6:**
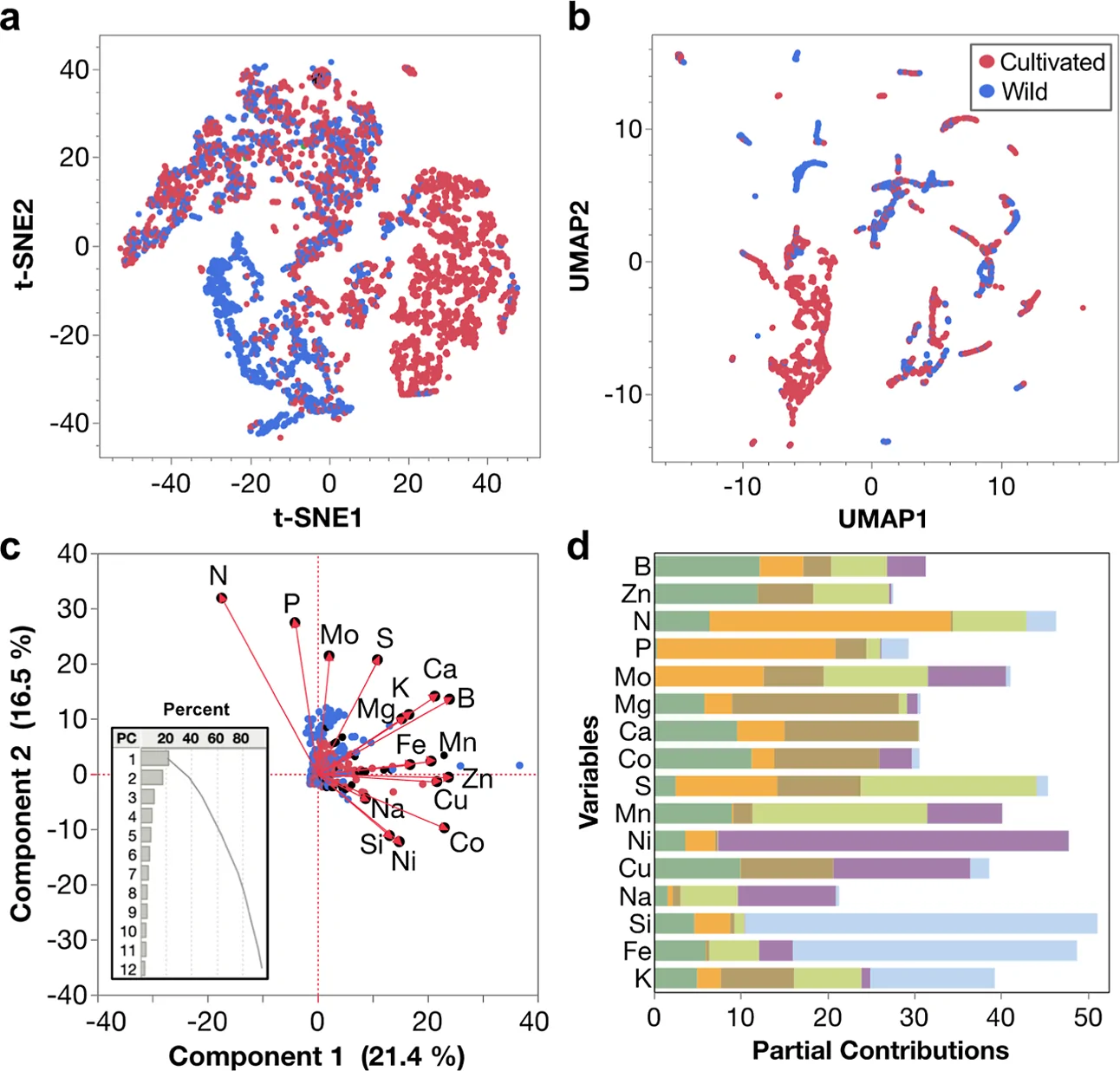
Multivariate analysis of the elements included in the study. (a) t-SNE projection of domesticated vs wild plants. (b) UMAP projection of domesticated and wild plants. (c) PCA biplot projection of domesticated vs nondomesticated plants including the scree plot of cumulative component explanation of total variance. (d) Partial contributions of the elements to overall principal coordinates, showing principal coordinates 1 (green) through 6 (purple) in order from left to right.

In the PCA (Figure 6c), the first two principal components (PC1 = 21.4% and PC2 = 16.5%) further indicate that cultivated plants plot with less variability than wild plants. The inset scree plot confirms that the first 6 PCs together explain ∼75% of the total variance. The partial contribution of these 6 variables (Figure 6d) indicate few readily explanable relationships, yet shows that each element has a specific contribution.Figure 6 The rank order of contributions may mirror contemporary fertilization regimes, further implicating agricultural management as the primary driver of stoichiometric convergence among cultivated plants.

Multivariate statistical frameworks such as PCA, t-SNE, and UMAP have long been used to delineate drivers of variability in plant nutrition traits, but almost all studies have focused on a small subset of macronutrients and common micronutrients.^28^ Analyses, including dozens of elements in concert, are almost absent, leaving the contribution of rare, nonessential, and potentially beneficial or toxic elements largely unknown. Recent trait database efforts^27^ still primarily address macronutrients, affirming the novelty of integrating trace and rare elements into compositional ordination for both domesticated and wild plants.

### Elemental Molar Ratios of Plant Elements

The molar element ratios of plants condense complex nutritional data into indicators that reflect core plant physiology, ecological strategies, and responses to environmental conditions. The molar element ratios were stratified by wild and domesticated plant categories and expressed as medians to minimize outlier effects (Table 5). The computed N:P molar ratio for wild plants (27.5) is at the upper end of published global leaf data,^12,13,33^ which is generally interpreted as an indicator of relatively stronger P-limitation, particularly in older or stress-prone plants. The K:Na ratio in wild plants (22.0) compared to domesticated plants (81.8) reflects selective breeding and management practices that enhance K uptake while minimizing Na accumulation. The Ca:Mg ratio in wild plants (1.3) compared to domesticated plants (0.5) indicates targeted fertilization and liming practices that maintain optimal Mg status in managed soils.^34^ The N:S ratio (wild: 9.6, domesticated: 24.8) shows that wild plants approach the global mean range (12–18), while domesticated plants exhibit elevated N:S, consistent with disproportionate N input and relatively lower S fertilization, a pattern important for protein synthesis and amino acid balance.

**Table 5:** Elemental Molar Ratios (mol:mol) of Select Stoichiometric Measures Using the Derived Global Plant Abundances

molar ratio			
	wild	domesticated	process/use
N:P	27.5	17.6	N- vs P-limitation, growth rate, homeostasis
K:N	22.0	81.8	salinity adaptation, ion selectivity
Ca:Mg	1.3	0.5	calcicole/calcifuge, soil adaptation
K:Mg	2.4	2.7	grass tetany risk, cation competition
N:S	9.6	24.8	protein synthesis, amino acid balance
Zn:Cd	248	4696	metal selectivity, phytoremediation
Fe:Mn	4.0	3.6	redox response, stress/tolerance
Si:Al	1.7	2.6	grass, Si-accumulation, plant defense
Mn:Fe	0.3	0.3	soil redox, metal balance
Ca:K	0.4	0.1	cation competition
S:Fe	11.4	41.1	Fe–S metabolism, stress signaling
La:Ce	0.6	0.5	rare earth provenance, potential fingerprint

Trace element ratios, such as Zn:Cd (wild: 248, domesticated: 4696) and Fe:Mn (wild: 4.0, domesticated: 3.6), span a wider range than literature expectations for plants on uncontaminated soils.^8^ The dramatic elevation of Zn:Cd in domesticated plants likely reflects rigorous food safety practices and selective exclusion of Cd through soil management and crop selection in addition to possible Zn fortification efforts. The Si:Al ratio (wild: 1.7, domesticated: 2.6) indicates an increased prevalence of silica-accumulating grasses or specialized species in the domesticated plant data set, as dicots rarely exceed ratios of 1.^7^ The REE ratio La:Ce (wild: 0.6, agricultural: 0.5) is lower than literature reports for studies that have examined REEs.^15^ The ratios provided here are a valuable baseline to compare future analyses of plant tissues against global averages.

### Identification of Hyperaccumulators

There were 53 unique species that met established thresholds for hyperaccumulation based on previous literature^15^ (Table 6 and Figure S2). The largest number of species were identified by Ni, followed by Cu, Pb, and Mn. Hyperaccumulators were represented by the phylogenetic families of 7 grasses (*Agrostis*, *Elymus*, *Melinis*, *Sporobolus*, *Paspalum*, *Cynodon*, and *Piptatherum*), 10 shrubs/small trees (*Acacia*, *Cytisus*, *Ulex*, *Erica*, *Leucaena*, *Peltophorum*, *Antidesma*, *Homalium*, *Glochidion*, and *Casearia*), and 5 herbs (*Bidens*, *Armeria*, *Plantago*, *Dichapetalum*, and *Phyla*). The species exhibit a broad range of ecological, functional, and geographic diversity. Table S2 includes hyperaccumulators that did not occur in native soils.

**Table 6:** Summary of Hyperaccumulator Plant Species Identified Exclusively from In Situ Collection Records for 9 Selected Elements (As, Cd, Co, Cr, Cu, Mn, Ni, Pb, and Zn) and Their Established Hyperaccumulation Threshold, the Major Plant Organ (Shoots or Roots) in Which Hyperaccumulation Was Observed, and the Total Number of Hyperaccumulator Species Detected (Counted Separately for Shoots and Roots)^a^

element	threshold (mg kg^–1^ DW)	organ	*n*	species
As	1,000	root	2	*Agrostis delicatula; Cytisus multiflorus*
		shoot	2	*Agrostis delicatula*; *Cytisus multiflorus*
Cd	100	shoot	1	*Lemna minor*
Co	300	shoot	4	*Aporosa chalarocarpa*; *Glochidion sericeum*; *Homalium kanaliense*; *Mischocarpus sundaicus*
Cr	300	shoot	4	*Anisophyllea disticha*; *Phyllanthus* sp.; *Psychotria sarmentosa*; *Sporobolus pyramidalis*
Cu	300	root	10	*Bidens alba*; *Cynodon dactylon*; *Erica arborea*; *Gentiana pennelliana*; *Lantana camara*; *Melinis repens*; *Paspalum notatum*; *Phyla nodiflora*; *Sporobolus pyramidalis*; *Ulex europaeus*
		shoot	9	*Cytisus multiflorus*; *Erica arborea*; *Lantana camara*; *Melinis repens*; *Mischocarpus sundaicus*; *Paspalum notatum*; *Pergularia tomentosa*; *Sporobolus pyramidalis*; *Ulex europaeus*
Mn	10,000	root	1	*Erica arborea*
		shoot	8	*Antidesma leucopodum*; *Antidesma coriaceum*; *Aporosa falcifera*; *Ardisia* sp.; *Erica arborea*; *Hedyotis* sp.; *Ilex oppositifolia*; *Urophyllum macrophyllum*
Ni	1,000	shoot	13	*Agatea longipedicillata*; *Aporosa chalarocarpa*; *Baccaurea lanceolata*; *Casearia silvana*; *Geissois pruinosa*; *Glochidion sericeum*; *Glochidion* sp.; *Homalium guillainii*; *Homalium kanaliense*; *Hybanthus austrocaledonicus*; *Phyllanthus securinegoides*; *Psychotria densifolia*; *Sebertia acuminata*
Pb	1,000	root	8	*Acacia mangium*; *Elymus macrourus*; *Eucalyptus camaldulensis*; *Leucaena floribunda*; *Leucaena leucocephala*; *Peltophorum pterocarpum*; *Phyla nodiflora*; *Piptatherum miliaceum*
		shoot	3	*Leucaena floribunda*; *Leucaena leucocephala*; *Ulex europaeus*
Zn	3,000	shoot	5	*Armeria maritima*; *Dichapetalum gelonioides*; *Glochidion* sp.; *Lemna minor*; *Plantago major*

a Hyperaccumulators are defined as species with concentrations equal to or greater than the element-specific threshold in the indicated organ, based on previously reported concentrations.^15^

Additional reviews highlight that hyperaccumulation is heavily skewed toward certain metals, climates, and taxa, with grass, shrub, and herb representation typical of both field surveys and global inventories.^8^ For trace elements, threshold definitions and systematic screening data remain unavailable; prior syntheses have called for trait-based approaches to catalog their environmental occurrence.^15,16^ Large global syntheses further report several hundred hyperaccumulator species spanning As, Cd, Cu, Cr, Mn, Ni, Pb, and Zn, demonstrating that hyperaccumulation is phylogenetically widespread and occurs on multiple continents and soil types.^36–39^ The identification in this study of 53 hyperaccumulator species thus represents a subset of this broader flora, restricted to cases where field observations in the database exceed established element-specific thresholds.

## Supplementary Material



## Data Availability

The database generated from this study is available as a spreadsheet in the Supporting Information. The database and all R code used to generate the analyses are available at https://github.com/MoonVillageAssociation/LAST-Plant_Nutrition.

## Notes

Systematic comparison of statistical models (fixed-effect vs mixed-effect); violin–box plots showing the distribution of concentrations for ten elements (As, Cd, Se, Co, Pb, Cr, Ni, Cu, Mn, and Zn); raw measures of database central tendency; summary of hyperaccumulator plant species; global LS-mean plant concentrations, continental crust reference concentrations used for comparison, and resulting molar accumulation ratios (PDF)
