# Supplementary material for: The Elemental Composition of Land Plants: A Global Database and Meta-analysis
Source: ACS Environ Au. 2026 Jul 23;6(5):814–28. doi: 10.1021/acsenvironau.6c00028 (PMC13602335; doi:10.1021/acsenvironau.6c00028)
Supplement: Supplementary file 1 [file vg6c00028_si_001.pdf]

Supporting information for publication:

The elemental composition of land plants: a global database and meta-analysis

Harrison R. Coker[1]
Aenghus C. Denvir[1]
Amir M. Mokhtari[2]
Anna Lewkowicz[3]
Patrick Grove[4]
Kristina Mikhailova[5]
Colin Lennox[6]
Marina López-Pozo[7]
Artemi Jaumà-Palomeras[8]
Luke G. Whiteley[1]
Rachel C. Rivero[9]
Borja Barbero Barcenilla[10]
Julie A. Howe[1*]

*Corresponding author: Julie A. Howe (j-howe@tamu.edu)

[1] Texas A&M University, Texas A&M AgriLife Research, Department of Soil and Crop Sciences, College Station, TX 77843, United States of America
[2] Polish Academy of Sciences, Department of Developmental Biology, Institute of Dendrology, Kórnik, 62-035, Poland
[3] ETH Zürich, Department of Environmental Systems Science, Zurich, 8092, Switzerland
[4] The Spring Institute for Forests on the Moon, Pleaux, 15700, France
[5] Cornell University, Department of Chemistry and Chemical Biology, Ithaca, NY 14853, United States of America
[6] EcoIslands LLC, Altoona, PA 16601, United States of America
[7] University of Colorado, Department of Ecology and Evolutionary Biology, Boulder, CO 80309, United States of America
[8] Universitat Politècnica de Catalunya, Barcelona, 08034, Spain
[9] University of Michigan, Department of Molecular, Cellular, and Developmental Biology, Ann Arbor, MI 48109, United States of America
[10] Department of Biochemistry and Biophysics, Texas A&M University, College Station, TX 77843, United States of America

Supporting information

Supporting file 1. The compiled database used to generate the current study.

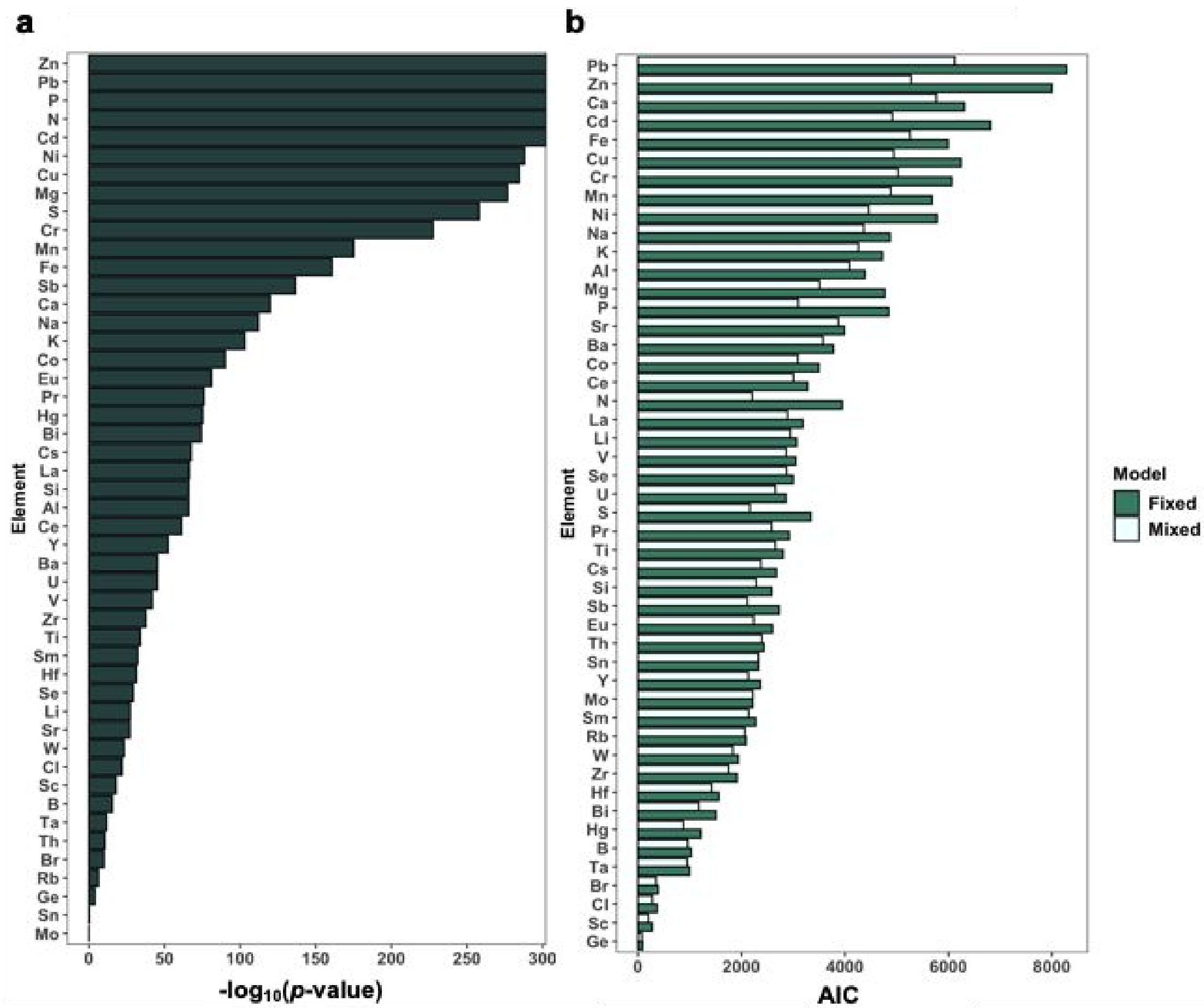

Supporting figure 1. Systematic comparison of statistical models (fixed-effect vs mixed-effect) to reveal the complexity of drivers behind each element's global concentration pattern, revealing whether deterministic group effects or unmeasured biological heterogeneity dominate nutrient variation across the plant kingdom. (a) The -log10(p-values) of statistical tests; all elements contained significant differences in linear prediction except Sn and Mo. (b) Information-theoretic metric reported as Akaike Information Criterion (AIC). Lower AIC values indicate better model fit. All elements contained improved mixed-effect predictions.

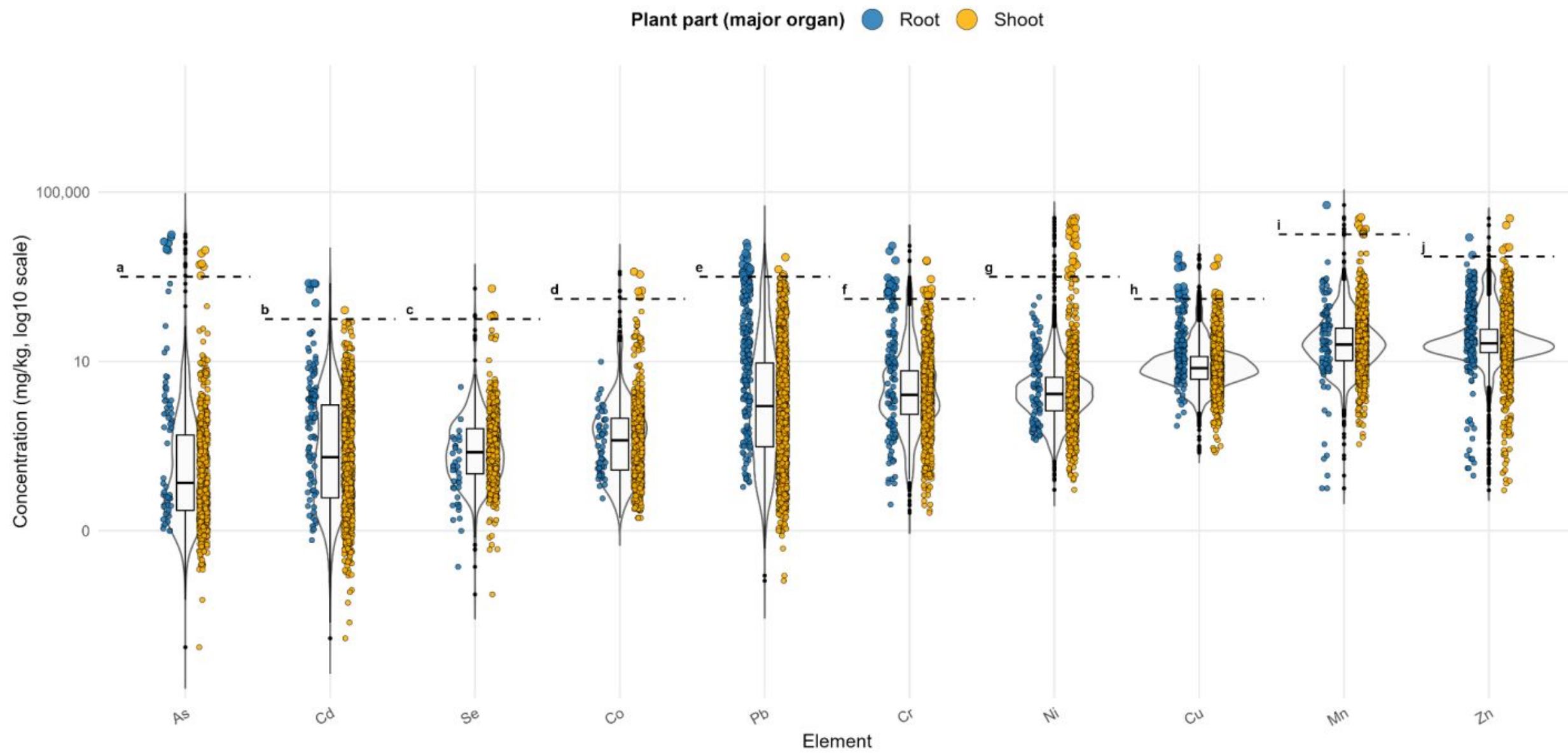

Supporting figure 2. Violin–box plots showing the distribution of concentrations for ten elements (As, Cd, Se, Co, Pb, Cr, Ni, Cu, Mn, Zn) in plant tissues, based on the entire dataset . Plant tissues are partitioned by major organ (shoots in orange, roots in blue). Data are shown on a $\log_{10}$ scale (mg/kg dry weight). For each element, a dashed horizontal line indicates the established hyperaccumulation threshold, and the letters a–j mark the thresholds in alphabetical order from left to right across the x-axis. Points represent individual species records; larger points denote values exceeding the corresponding threshold, i.e., hyperaccumulator cases. Unknown organ assignments were excluded from the analysis.

Supporting table 1: Measures of central tendency of the database.

Element	*n*	mean	median	q25	q75	IQR
N	1581	32342	22700	16700	29200	12500
K	1498	14699	10700	3980	18259	14279
Ca	1411	6062	2500	351	8980	8630
S	1289	5736	5220	3620	7270	3650
P	1421	3344	2540	1420	3840	2420
Cl	92	3276	2255	852	4763	3911
Mg	1502	2793	2012	1170	3500	2330
Na	1060	1326	123	21	552	531
Si	647	1033	127	45	475	430
Al	1016	638	48	13	239	226
Fe	1585	348	95	36	273	237
Mn	1517	199	25.2	10.5	61.5	51.0
Ni	1410	167	1.7	0.7	4.2	3.5
Zn	2147	126	26.6	16.1	57.0	40.9
Sc	53	109	8.0	0.2	90.0	89.8

As	726	79.18	0.01	0.00	0.18	0.18
Pb	1592	70.11	0.88	0.10	9.20	9.11
Br	108	53.25	17.40	6.44	73.00	66.56
Sr	940	44.36	9.73	1.96	46.45	44.49
Ti	639	38.97	1.79	0.51	11.50	10.99
Ba	877	33.73	7.06	1.02	32.30	31.28
Cr	1402	32.71	1.64	0.56	6.00	5.44
Cu	1951	27.06	6.95	3.80	13.04	9.24
Nd	534	26.06	0.10	0.01	0.73	0.71
Ce	668	24.95	0.20	0.03	1.22	1.19
Rb	612	22.77	9.22	3.62	25.93	22.31
La	639	21.72	0.11	0.01	0.84	0.83
B	287	16.70	11.00	2.55	25.45	22.90
Y	476	15.55	0.044	0.007	0.381	0.373
Cs	600	8.40	0.013	0.004	0.056	0.053
Pr	583	8.00	0.051	0.003	0.296	0.293
Co	812	7.23	0.139	0.027	0.454	0.427
Cd	1357	5.28	0.056	0.006	0.940	0.934
Eu	506	4.55	0.004	0.000	0.026	0.025
V	657	3.02	0.287	0.032	1.410	1.378
Se	742	2.71	0.073	0.022	0.259	0.237
Sm	480	2.42	0.014	0.002	0.102	0.099
U	593	2.40	0.002	0.000	0.020	0.019
Bi	312	2.34	0.001	0.000	0.004	0.003
Mo	612	2.34	0.504	0.202	1.213	1.011
Zr	414	2.12	0.043	0.008	0.151	0.143
Yb	480	1.77	0.004	0.001	0.024	0.023
Li	741	1.49	0.123	0.029	0.400	0.371
Tb	480	1.25	0.001	0.000	0.011	0.010
Hg	261	1.21	0.026	0.009	0.147	0.138
Sn	583	1.14	0.053	0.015	0.151	0.136
Th	546	1.11	0.010	0.002	0.064	0.062
Sb	628	1.09	0.002	0.001	0.008	0.007
Ge	27	1.06	0.036	0.019	0.104	0.085
Hf	366	1.02	0.002	0.000	0.005	0.005
W	537	1.01	0.004	0.002	0.010	0.008
Ta	277	1.00	0.002	0.001	0.004	0.003

Supporting table 2. Summary of hyperaccumulator plant species identified across the entire dataset for 10 selected elements. The table presents the established hyperaccumulation threshold, the major plant organ (shoots or roots) in which hyperaccumulation was observed, the total number of hyperaccumulator species detected (counted separately for shoots and roots), and the corresponding species names. Hyperaccumulators are defined as species with concentrations equal to or greater than the element-specific threshold in the indicated organ, based on concentrations reported by (van der Ent et al., 2013)

Element	Threshold (mg/kg DW)	Organ	#	Species names
As	1000	Root	3	*Acacia farnesiana*; *Agrostis delicatula*; *Cytisus multiflorus*
		Shoot	3	*Acacia farnesiana*; *Agrostis delicatula*; *Cytisus multiflorus*
Cd	100	Root	1	Acanthus ilicifolius
		Shoot	1	Lemna minor
Co	300	Shoot	4	*Aporosa chalarocarpa*; *Glochidion sericeum*; *Homalium kanaliense; Mischocarpus sundaicus*
Cr	300	Root	2	*Helianthus annuus*; *Prunus persica*
		Shoot	7	*Anisophyllea disticha*; *Eleusine coracana*; *Helianthus annuus*; *Phyllanthus sp.*; *Prunus persica*; *Psychotria sarmentosa*; *Sporobolus pyramidalis*
Cu	300	Root	11	*Bidens alba*; *Cynodon dactylon*; *Erica arborea*; *Gentiana pennelliana*; *Lantana camara*; *Melinis repens*; *Paspalum notatum*; *Phyla nodiflora*; *Sporobolus pyramidalis*; *Ulex europaeus*; *Zea mays*
		Shoot	10	*Cytisus multiflorus*; *Erica arborea*; *Lantana camara*; *Melinis repens*; *Mischocarpus sundaicus*; *Paspalum notatum*; *Pergularia tomentosa*; *Sporobolus pyramidalis*; *Ulex europaeus*; *Zea mays*

Mn	10000	Root	1	Erica arborea
		Shoot	8	*Antidesma leucopodum*; *Antidesma coriaceum*; *Aporosa falcifera; Ardisia sp.*; *Erica arborea; Hedyotis sp.*; *Ilex oppositifolia; Urophyllum macrophyllum*
Ni	1000	Shoot	13	*Agatea longipedicillata*; *Aporosa chalarocarpa*; *Baccaurea lanceolata*; *Casearia silvana*; *Geissois pruinosa*; *Glochidion sericeum*; *Glochidion sp.* ; *Homalium guillainii*; *Homalium kanaliense*; *Hybanthus austrocaledonicus*; *Phyllanthus securinegoides*; *Psychotria densifolia*; *Sebertia acuminata*
Pb	1000	Root	13	*Acacia mangium*; *Calendula arvensis*; *Cynodon dactylon*; *Elymus macrourus*; *Eucalyptus camaldulensis*; *Leucaena floribunda*; *Leucaena leucocephala*; *Peltophorum pterocarpum*; *Phyla nodiflora*; *Piptatherum miliceum*; *Polygonum persicarium*; *Solanum nigrum*; *Sylibum marianum*
	1000	Shoot	4	*Cichorium intybus*; *Leucaena floribunda*; *Leucaena leucocephala*; *Ulex europaeus*
Se	100	Shoot	4	*Capsicum frutescens*; *Cymbopogon nervatus*; *Pennisetum glaucum*; *Ziziphus spina*-christi
Zn	3000	Root	2	*Calendula arvensis*; *Sylibum marianum*
	3000	Shoot	5	*Armeria maritima*; *Dichapetalum gelanioides*; *Glochidion sp.* ; *Lemna minor* ; *Plantago major*

Supporting table 3. Global LS-mean plant concentrations, continental crust reference concentrations used for comparison, and resulting accumulation ratios for all analyzed elements. Reference abundances used in the accumulation-ratio analysis represent published global crustal

benchmarks rather than measured soil concentrations at each plant sampling location; therefore, the ratios are intended for broad comparative interpretation only.

Element	Terrestrial abundance (mg/kg)	Plant abundance (mg/kg)	Accumulatio ratio
N	83	14059	169.385542
S	62	1253	20.2096774
Br	1.6	13.2	8.25
Cd	0.09	0.39	4.33333333
Se	0.09	0.37	4.11111111
P	655	1188	1.81484876
Bi	0.16	0.18	1.125
Cl	370	388	1.04864865
B	17	11.4	0.67058824
Zn	67	33	0.49253731
Hg	0.05	0.02	0.4
Mo	1.1	0.42	0.38181818
Cu	28	9.89	0.35321429
K	23243	7075	0.30439534
Sb	0.4	0.09	0.225
As	4.8	0.81	0.16875
Pb	17	2.78	0.16352941
Cs	4.9	0.51	0.10408163
Mg	14954	1556	0.10404964
Sc	14	1.2	0.08571429
Ca	25657	2124	0.08278207
Ni	47	3.59	0.07638298
Eu	1	0.07	0.07
Pr	7.1	0.403	0.05681277
Mn	774	42.9	0.05539057
Rb	84	4.61	0.05488095
Y	21	1.1	0.05238095
La	31	1.54	0.04967742
Sr	320	15.2	0.0475
Co	17.3	0.6	0.03468208
Sm	4.7	0.14	0.02978723
Cr	92	2.64	0.02869565

Ge	1.4	0.04	0.02857143
Sn	2.1	0.04	0.01904762
V	97	1.34	0.01381443
Li	24	0.3	0.0125
Na	24260	300	0.01236597
Ba	628	7.31	0.01164013
Ta	0.9	0.01	0.01111111
Ce	63	0.66	0.01047619
Hf	5.3	0.04	0.00754717
Ti	3836	27.9	0.0072729
Th	10.5	0.07	0.00666667
W	1.9	0.01	0.00526316
Zr	193	0.6	0.00310881
Fe	45349	139	0.00306506
Al	81512	110	0.00134949
Si	311288	240	0.00077099
